# Supplementary material for: Development and Efficacy Evaluation of a Novel Nanoparticle-Based Hemagglutination Inhibition Assay for Serological Studies of Porcine Epidemic Diarrhea Virus
Source: Vet Sci. 2025 Feb 1;12(2):101. doi: 10.3390/vetsci12020101 (PMC11861430; doi:10.3390/vetsci12020101)
Supplement: Supplementary file 1 [file vetsci-12-00101-s001.zip › vetsci-3375094-supplementary.pdf]

Supplementary Table S1. HI and VN titer of 54 positive samples detected by HI assay

Serum No.	Titer of VN test	Titer of HI assay
1	1: 128	320
2	1: 128	160
3	1: 64	160
4	1: 64	80
5	1: 128	320
6	1: 32	80
7	1: 64	160
8	1: 256	640
9	1: 128	320
10	1: 128	320
11	1: 16	80
12	1: 128	320
13	1: 128	160
14	1: 32	80
15	1: 64	160
16	1: 64	160
17	1: 32	160
18	1: 16	80
19	1: 16	80
20	1: 128	320
21	1: 32	80
22	1: 32	80
23	1: 256	320
24	1: 256	640
25	1: 16	80
26	1: 256	640
27	1: 64	160
28	1: 256	640
29	1: 16	80
30	1: 32	160
31	1: 16	80
32	1: 256	640
33	1: 64	160
34	1: 128	320
35	1: 16	80
36	1: 32	160
37	＜1: 8	80
38	＜1: 8	80
39	＜1: 8	80
40	＜1: 8	80
41	＜1: 8	80
42	＜1: 8	80
43	＜1: 8	80
44	＜1: 8	80
45	＜1: 8	80
46	＜1: 8	80
47	＜1: 8	80
48	＜1: 8	80
49	＜1: 8	80
50	＜1: 8	80
51	＜1: 8	80
52	＜1: 8	80
53	＜1: 8	160
54	＜1: 8	160
