# Supplementary figures and images for: Development and Efficacy Evaluation of a Novel Nanoparticle-Based Hemagglutination Inhibition Assay for Serological Studies of Porcine Epidemic Diarrhea Virus
Source: Vet Sci. 2025 Feb 1;12(2):101. doi: 10.3390/vetsci12020101 (PMC11861430; doi:10.3390/vetsci12020101)

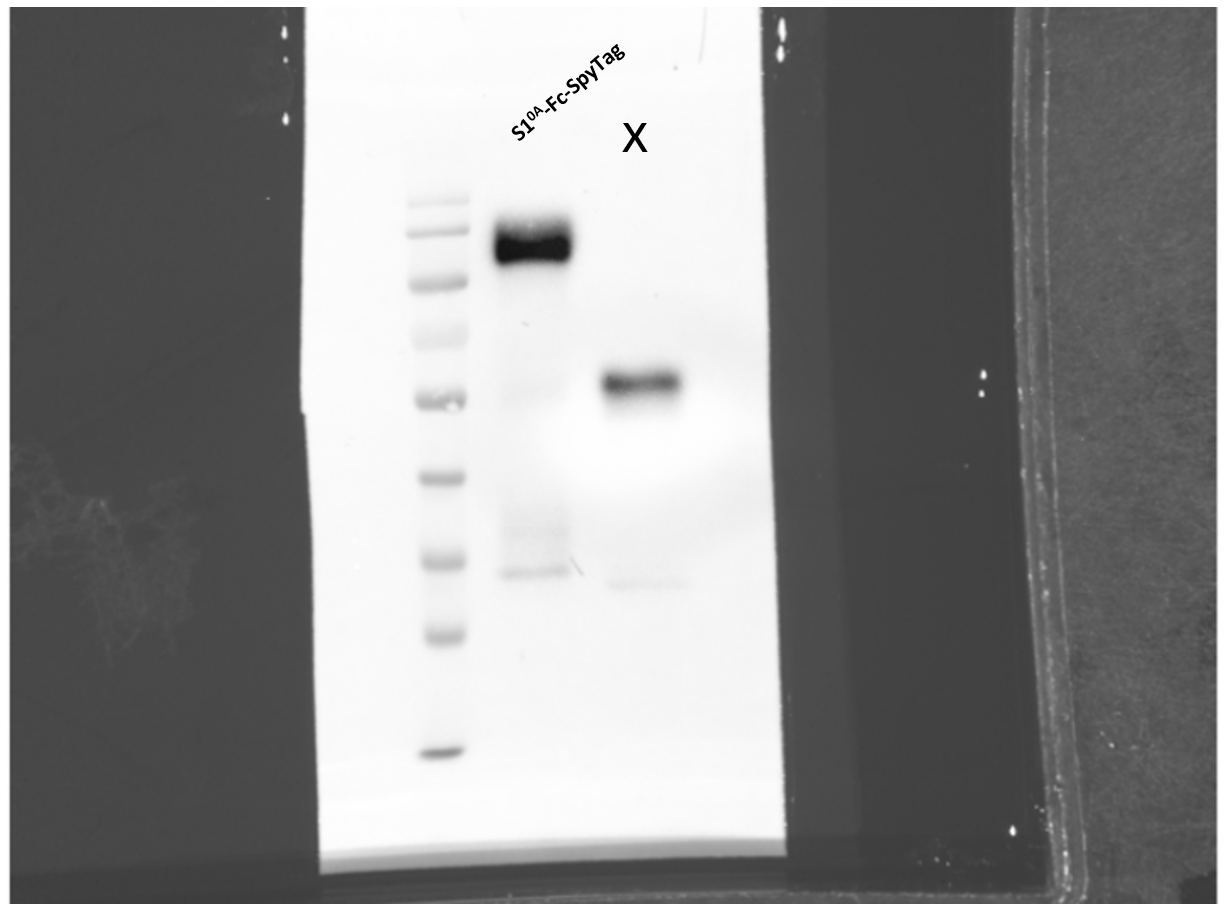

Supplement: Supplementary file 1 [file vetsci-12-00101-s001.zip › Original Images/Fig1B.Affinity-purified Fc-tagged S10A-Spy was identified by Western blot. M, protein marker, with numbers on the left side indicate molecular masses (in kilodaltons).tif]

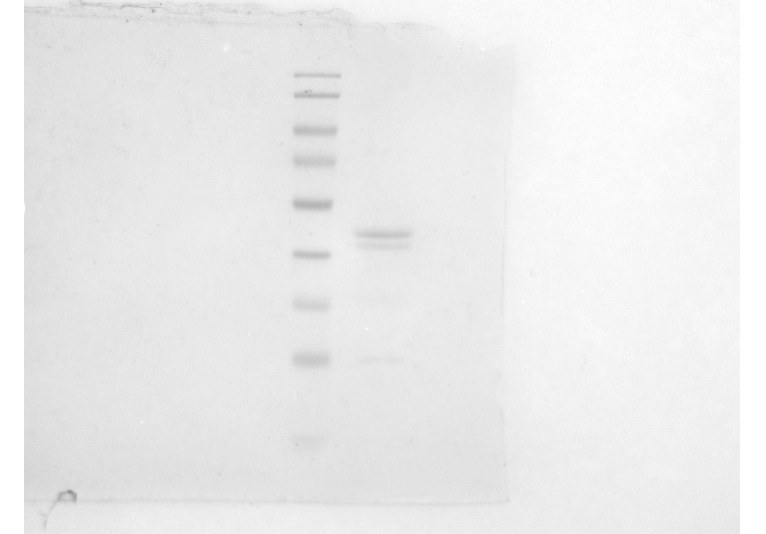

Supplement: Supplementary file 1 [file vetsci-12-00101-s001.zip › Original Images/Fig1D.Purified NPs were analyzed by SDS-PAGE. M is a protein marker, Numbers on the left are molecular masses (in kilodaltons).tif]

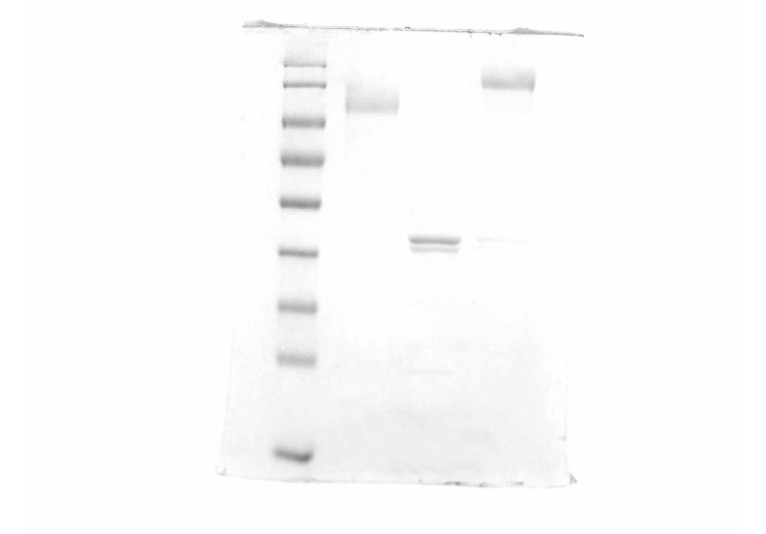

Supplement: Supplementary file 1 [file vetsci-12-00101-s001.zip › Original Images/Fig2A.SpyCatcher-mi3 was incubated with S10A-SpyTag at a 1.5,1 molar ratio at 25°C for 36 h, followed by analysis of S10A-NP formation via reducing SDS-PAGE.tiff]

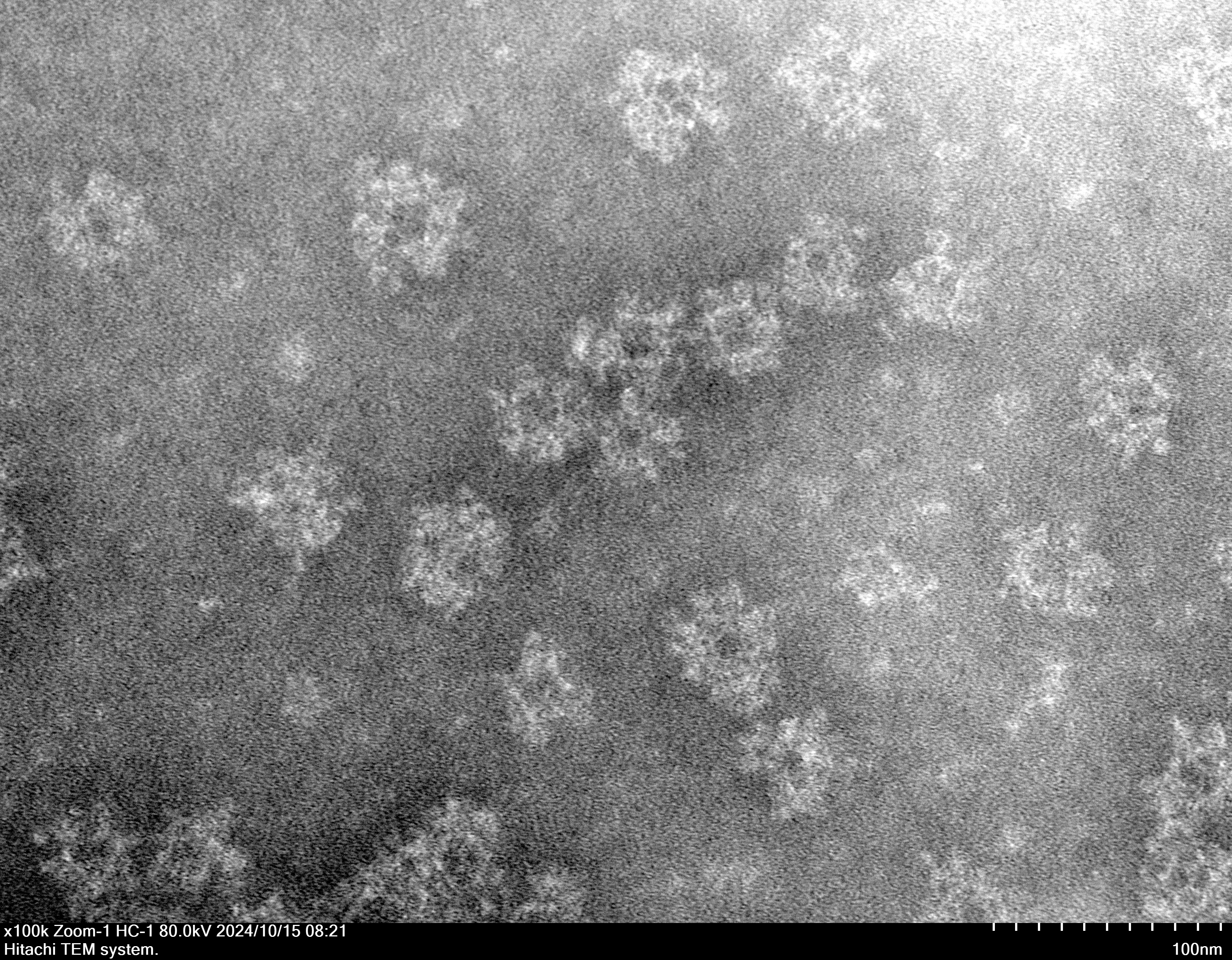

Supplement: Supplementary file 1 [file vetsci-12-00101-s001.zip › Original Images/Fig2B. TEM image of S10A-NPs was negatively stained. Scale bar 100nm. .tif]
